# Neonatal encephalopathy due to suspected hypoxic ischemic encephalopathy: pathophysiology, current, and emerging treatments

**DOI:** 10.1007/s12519-024-00836-9

**Published:** 2024-09-06

**Authors:** Carina Corte-Real Babbo, Juanita Mellet, Jeanne van Rensburg, Shakti Pillay, Alan Richard Horn, Firdose Lambey Nakwa, Sithembiso Christopher Velaphi, Gugulabatembunamahlubi Tenjiwe Jabulile Kali, Melantha Coetzee, Mogomane Yvonne Khomotso Masemola, Daynia Elizabeth Ballot, Michael Sean Pepper

**Affiliations:** 1 SAMRC Extramural Unit for Stem Cell Research and Therapy, Department of Immunology, Faculty of Health Sciences, Institute for Cellular and Molecular Medicine, University of Pretoria, Room 5-64, Level 5, Pathology Building, 15 Bophelo Road (Cnr. Steve Biko and Dr. Savage Streets), Prinshof Campus, Gezina, Pretoria, South Africa; 2 Department of Paediatrics and Child Health, Division of Neonatology, Groote Schuur Hospital, University of Cape Town, Neonatal Unit, Cape Town, South Africa; 3 Department of Paediatrics and Child Health, Faculty of Health Sciences, Chris Hani Baragwanath Academic Hospital, University of the Witwatersrand, Johannesburg, South Africa; 4 Department of Paediatrics and Child Health, Stellenbosch University, Tygerberg Hospital Neonatal Unit, Cape Town, South Africa; 5 Department of Paediatrics and Child Health, Division of Neonatology, Faculty of Health Sciences, Steve Biko Academic Hospital, University of Pretoria, Pretoria, South Africa; 6 Department of Paediatrics and Child Health, Faculty of Health Sciences, Kalafong Hospital, University of Pretoria, Pretoria, South Africa; 7 Department of Paediatrics and Child Health, Faculty of Health Sciences, Charlotte Maxeke Johannesburg Academic Hospital, University of the Witwatersrand, Johannesburg, South Africa

**Keywords:** Brain, Cerebrovascular circulation, Hypoxia–ischemia, Induced hypothermia, Neuroprotective agents

## Abstract

**Background:**

Neonatal encephalopathy (NE) due to suspected hypoxic-ischemic encephalopathy (HIE), referred to as NESHIE, is a clinical diagnosis in late preterm and term newborns. It occurs as a result of impaired cerebral blood flow and oxygen delivery during the peripartum period and is used until other causes of NE have been discounted and HIE is confirmed. Therapeutic hypothermia (TH) is the only evidence-based and clinically approved treatment modality for HIE. However, the limited efficacy and uncertain benefits of TH in some low- to middle-income countries (LMICs) and the associated need for intensive monitoring have prompted investigations into more accessible and effective stand-alone or additive treatment options.

**Data sources:**

This review describes the rationale and current evidence for alternative treatments in the context of the pathophysiology of HIE based on literatures from Pubmed and other online sources of published data.

**Results:**

The underlining mechanisms of neurotoxic effect, current clinically approved treatment, various categories of emerging treatments and clinical trials for NE are summarized in this review. Melatonin, caffeine citrate, autologous cord blood stem cells, Epoetin alfa and Allopurinal are being tested as potential neuroprotective agents currently.

**Conclusion:**

This review describes the rationale and current evidence for alternative treatments in the context of the pathophysiology of HIE. Neuroprotective agents are currently only being investigated in high- and middle-income settings. Results from these trials will need to be interpreted and validated in LMIC settings. The focus of future research should therefore be on the development of inexpensive, accessible monotherapies and should include LMICs, where the highest burden of NESHIE exists.

**Graphical abstract:**

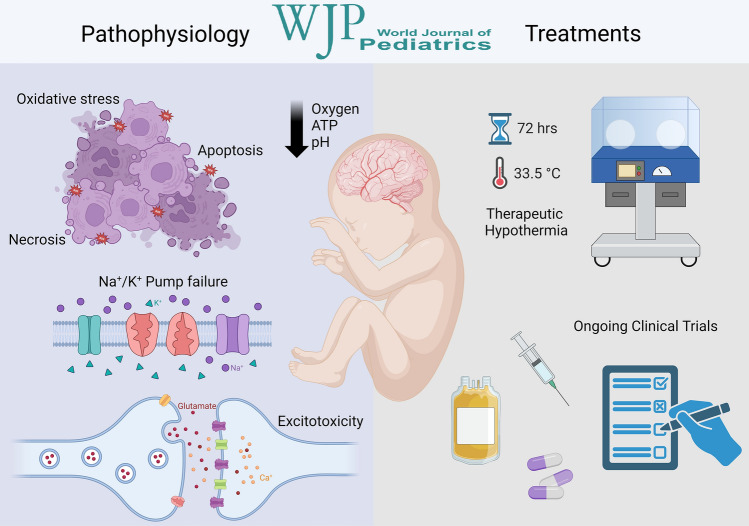

## Introduction

Neonatal encephalopathy (NE) is a non-specific clinical syndrome of disturbed neurological function in late preterm or term newborns, characterized by an altered level of consciousness, seizures, poor tone, and difficulty in initiating and maintaining respiration [1]. There are many causes of NE including neonatal stroke, epileptic encephalopathies, infections, metabolic disorders, and placental abnormalities [2]; however, in the majority of cases, the cause is unexplained. When NE occurs in babies following an acute peripartum or intrapartum event that impedes cerebral blood flow and fetal oxygen delivery (hypoxia–ischemia), the cause of NE is considered to be hypoxic-ischemic encephalopathy (HIE) [3, 4]. The term NESHIE is used to refer to NE due to suspected HIE. NESHIE is a diagnosis inferred from non-specific clinical and metabolic criteria, including low Apgar scores, acidaemia on umbilical cord blood (UCB) or early infant blood gas within the first hour of birth, and a history of a sentinel event in close temporal proximity to labour and delivery [5–7]. Antenatal risk factors for NESHIE, such as nulliparity, maternal age above 35 years, gestation over 41 weeks, intrauterine growth restriction, and maternal urinary tract infections are associated with poor placental function and/or intrapartum compromise [2, 8]. The perinatal risk factors associated with HIE are those that may cause or be caused by acute peripartum hypoxia, such as cord prolapse, uterine rupture, placental rupture, prolonged second stage of labour, shoulder dystocia, and abnormal fetal heart rate [2, 8]. The grading system described by Sarnat and Sarnat delineates the degree of encephalopathy by categorizing the affected baby as having mild (Sarnat stage 1), moderate (Sarnat stage 2), or severe (Sarnat stage 3) HIE [9]. The severity of HIE is dependent on the severity of the hypoxic-ischemic event, additional risk factors, and ultimately the individual neonatal response. Babies with moderate encephalopathy have a 10% risk of death, with 30% of babies surviving with neurodevelopmental impairment. Meanwhile, those with severe encephalopathy face a 60% mortality rate, with almost all survivors having some degree of severe neurological impairment [10, 11].

The global incidence of HIE varies within and between countries [12–14]. Of the one million deaths that are attributed to HIE annually, the majority occur in low- to middle-income countries (LMICs) [15]. South African studies have shown wide variability in the incidence of HIE, depending on defining criteria and setting. A hospital-based study at a tertiary academic center in Johannesburg reported incidences ranging from 8.5 to 13.3 per 1000 live births [16]. In contrast, a population-based study from the Southern Cape Peninsula, recording admissions to one tertiary-level hospital and two secondary-level hospitals in Cape Town, reported incidences between 2.3 and 4.3 per 1000 live births [17].

## Pathophysiology

New and effective treatment strategies must be considered in the context of the complex and evolving pathophysiology of HIE. Apart from the potential permanent brain injury, the biochemical and histopathological consequences of the initial hypoxic injury can persist and affect the neonatal brain for weeks and even months afterward.

## Mechanisms and phases of energy failure and neurotoxic cascade

**Fig. 1:**
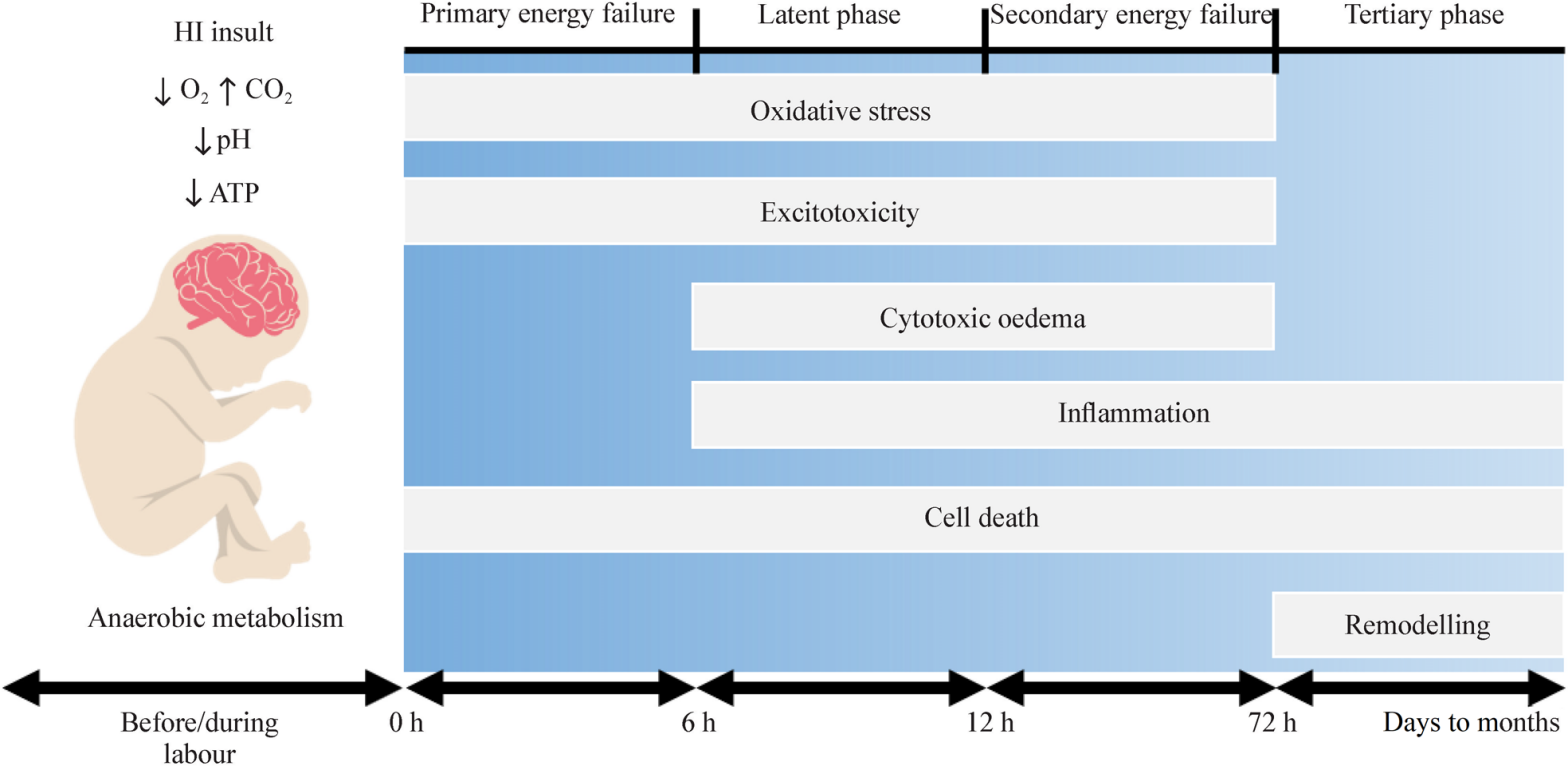
Phases and relative mechanisms of injury in HIE. Acute peripartum HI insult triggers a reduction in cerebral oxygen delivery, elevation in carbon dioxide levels, and decrease in blood pH and cellular ATP. *HIE* hypoxic-ischemic encephalopathy, *ATP* adenosine triphosphate, *CO*_*2*_ carbon dioxide, *HI* hypoxic-ischemic, *O*_*2*_ oxygen. Image adapted from Pedroza-Garcia, Calderon-Vallejo et al. [18]

The primary mechanisms of HIE-induced injury to the neonatal brain include (1) excitotoxicity, (2) oxidative stress, and (3) inflammation, which collectively contribute to neuronal cell death either via apoptosis or necrosis [18]. The injury can be divided into four phases following a hypoxic-ischemic (HI) insult just before, during, or immediately after labor (peripartum period): (1) primary energy failure phase (0–6 hours after) followed by (2) the latent phase (6–12 hours post the HI insult), (3) the secondary energy failure phase (12–72 h post the HI insult), if homeostasis is not effectively restored, and (4) the tertiary phase, where neuronal cell death may continue days to months after the initial injury (Fig. 1) [18].

The primary energy failure phase, which occurs within the first 6 hours following the HI insult, is characterized by a switch in cellular metabolism from aerobic to anaerobic; anaerobic metabolism is substantially less efficient in the production of adenosine triphosphate (ATP) [12, 19]. As a consequence of decreased ATP, the sodium–potassium pumps in the cell fail, leading to the intracellular accumulation of sodium ions (Na^+^), calcium ions (Ca^2+^), and water in the neural cells. The resulting membrane depolarization leads to the release of excitatory neurotransmitters, such as glutamate [12, 18]. Excessive release of glutamate and other excitatory neurotransmitters results in the overstimulation of the α-amino-3-hydroxy-5-methyl-4-isoxazolepropionic acid (AMPA), N-methyl-D-aspartate (NMDA), and kainate receptors [18]. This process of excitotoxicity is associated with oxidative stress, cytotoxic edema, activation of pro-apoptotic pathways, and ultimately cellular necrosis and/or accelerated apoptosis [20].

## Therapeutic hypothermia

The only evidence-based and clinically approved treatment that is currently available and used globally for moderate and severe HIE is therapeutic hypothermia (TH). A 2010 Cochrane review showed that initiating TH within 6 h of birth for babies with moderate to severe HIE significantly decreased mortality and major disability from 61 to 46% [21]. However, despite the significant reduction in mortality associated with the use of TH, many cooled babies with moderate to severe HIE still survive with disabilities [21, 22].

In 2021, the results of a large, multicentre randomized controlled trial (RCT) in India, Sri Lanka, and Bangladesh—the Hypothermia for Encephalopathy in Low- and Middle-Income Countries (HELIX) trial—showed that TH failed to decrease neurodevelopmental impairment and notably increased mortality from 31% to 42%. The authors concluded that all LMICs should immediately suspend the use of TH in babies with HIE [22]. In response, several research groups raised concerns regarding this conclusion [23–26]. The setting of the HELIX trial is not generalizable to all LMICs and its findings contradict other studies from high- and LMICs [27–32]. Diggikar and colleagues [33] analyzed data from 14 publications on TH in LMICs and concluded that TH is beneficial in reducing mortality and morbidity. However, the authors noted that the majority of the RCTs did not use servo-controlled cooling equipment (*n* = 10). Consequently, the study’s conclusion carries low certainty due to variability in the TH techniques and methods used in LMICs. The authors stated that TH may be harmful if supportive care measures are not optimal. A meta-analysis of ten RCTs from LMICs did not find any clear evidence of the benefit or harm associated with TH, with variability in the cooling equipment used [34].

Clinical trials have assessed the effectiveness of commencing TH after six hours of age [35]. Other studies have investigated adjusting the depth and duration of cooling to improve efficacy [36, 37]. There are currently no published studies showing that cooling babies after 6 hours of age or for longer than 72 hours at lower temperatures is beneficial [36, 37].

One challenge faced in LMICs is the high cost of automated servo-controlled cooling equipment. South African clinicians have achieved significant cost reduction by validating a servo-assisted gel-pack cooling method [38, 39]. However, this method still requires qualified staff, expertise, monitoring, supportive care, and adherence to good governance principles during TH.

In addition to the limitations of TH, the significant diversity among LMICs, in terms of infrastructure, socioeconomic conditions, healthcare service delivery, and cultural diversity [23] prevents the generalization of study findings to all LMICs. In addition, there are no other proven neuroprotective strategies for the management of HIE. Rather than abandoning TH as a neuroprotective treatment modality, the immediate priority should be to find cost-effective, reliable, and accessible adjuvant therapies that can be used in combination with TH or as monotherapy, especially for LMICs with the greatest HIE burden. Several clinical trials are currently underway testing the efficacy of various neuroprotective agents as adjuvant therapies to TH.

## Emerging treatments

There are several biological treatment modalities currently being tested as potential neuroprotective agents in babies with HIE. Table 1 shows agents currently being tested in clinical trials registered on ClinicalTrials.org.

**Table 1:** Neuroprotective agents being tested for the treatment of HIE

National clinical trial (NCT) number	Neuroprotective agent	Clinical trial phase	Start date	Estimated end date	Location
NCT02621944	Melatonin	Early phase 1	Nov 2016	Mar 2025	United States of America
NCT03913221	Caffeine citrate	Phase 1	July 2019	Dec 2024	United States of America
NCT02551003	Autologous cord blood	Phase 1, 2	Sep 2015	Dec 2025	China
NCT02881970	Autologous cord blood stem cells	Phase 1, 2	Feb 2020	Sep 2025	France
NCT03079167	Epoetin alfa	Phase 3	May 2016	Mar 2023	Australia, New Zealand, Singapore
NCT03162653	Allopurinol	Phase 3	Mar 2018	Jan 2026	Austria, Belgium, Estonia, Finland, Germany, Italy, Netherlands

*HIE* hypoxic-ischemic encephalopathy

## Melatonin

Melatonin (N-acetyl-5-methoxytryptamine) is a hormone produced by the pineal gland that regulates the circadian rhythm. Its role in neuroprotection is related to its anti-oxidant, anti-excitatory, anti-inflammatory, and anti-apoptotic properties [40, 41]. In addition, melatonin easily crosses the blood–brain barrier (BBB), has a good safety profile, and its metabolism is unaffected by TH [40, 42].

Melatonin is a potent reactive oxygen species (ROS) scavenger with antioxidant properties. After metabolism, it produces two powerful antioxidant molecules, namely N1-acetyl-N2-formyl-5-methoxykynuramine (AFMK) and N1-acetyl-5-methoxykynuramine (AMK) [43]. Melatonin blocks caspase-3 cleavage, which results in the degradation of cellular components and prevents the mitochondrial permeability transition pore from opening, thereby stabilizing the mitochondrial membrane and contributing to melatonin’s anti-apoptotic properties [44]. Anti-inflammatory properties are achieved through the suppression of pro-inflammatory cytokines, such as interleukin (IL)-1 and IL-8, tumor necrosis factor (TNF), cyclooxygenase (COX), and inducible nitric oxide synthase (iNOS) [44, 45]. In addition, melatonin inhibits the activation of the nucleotide-binding oligomerization domain (NOD)-like receptor family, nucleotide-binding domain, leucine-rich–containing family, pyrin domain-containing protein 3 (NLRP3) inflammasome, which triggers caspase 1 activation, cytokine release, and pyroptosis [44].

A randomized controlled pilot study in 2015 showed the feasibility and efficacy of early administration of melatonin, as a neuroprotective agent, to full-term neonates with HIE, managed with and without TH [40]. Participants received 10 mg/kg daily for 5 days. Melatonin was most effective in combination with TH, significantly decreasing levels of the superoxide dismutase (SOD) enzyme and nitric oxide (NO). High levels of both SOD and NO have previously been associated with severe HIE [46, 47]. The melatonin/hypothermia group had fewer seizures on follow-up electroencephalogram (EEG) and fewer white matter abnormalities on neuroimaging compared to the TH-only group. In 2018, a separate small randomized controlled trial showed improved survival rates at day 28 among 40 neonates who received melatonin (10 mg) in combination with TH [48]. In 2019, another randomized trial further tested melatonin’s neuroprotective capacity in combination with magnesium sulfate (MgSO_4_) in 30 neonates with moderate HIE. The study measured serum concentrations of S100-B, a marker for brain injury, and found lower levels in the combination therapy group compared to the melatonin-only group [49]. The above-mentioned studies only focused on short-term outcomes. More recently, in 2020, the results of a pilot study assessing the neuroprotective effects of melatonin in combination with TH showed improved cognitive scores at 18 months of age using the Bayleys III assessment [50]. Although these are seemingly positive outcomes, there remains insufficient evidence to conclude that melatonin can be used as a neuroprotective agent for HIE neonates. Long-term neurodevelopmental data are lacking, and more evidence is needed to ascertain any significant reduction in mortality rates [51, 52].

At present, a larger, non-randomized, dose escalation clinical trial (NCT02621944; Table 1) is underway to evaluate the neuroprotective effects and appropriate dosage of melatonin in combination with TH for the treatment of babies diagnosed with HIE. The results of this trial are expected in 2025.

## Caffeine citrate

Caffeine citrate is commonly administered to premature babies for the prevention and treatment of apnoea of prematurity. Current evidence supports the benefit of caffeine in reducing the frequency of apnoea, intermittent hypoxia, and extubation failure. The use of caffeine in neonates is deemed safe and may have long-term beneficial effects [53].

However, there is limited data and ongoing debate on the safety and efficacy of high-dose caffeine, optimal duration and initiation of prophylactic caffeine, and the value of caffeine in late preterm and term babies [54]. There are three proposed mechanisms of action for caffeine: (1) calcium mobilization, (2) phosphodiesterase inhibition, and (3) adenosine receptor antagonism; by blocking adenosine receptors in the brain [55]. In addition, the antioxidant and anti-inflammatory properties of caffeine may contribute to its neuroprotective effect.

The Caffeine for Apnea of Prematurity (CAP) trial showed that early administration of caffeine was associated with a lower incidence of bronchopulmonary dysplasia and severe retinopathy of prematurity [56]. Follow-up studies at 18 months, 5 years, and 11 years (NCT00182312) showed no long-term adverse outcomes. At 18–21 months of age, babies in the caffeine group had a lower incidence of cerebral palsy (CP) and cognitive delay [54]. At five years of age, the rates of death, motor impairment, behavioral problems, deafness, and blindness were not significantly different between the caffeine group and the control group. However, a secondary analysis showed that gross motor function was better and the incidence of developmental coordination disorder was reduced in the caffeine group. While caffeine did not markedly reduce the short-term rate of academic, motor, or behavioral impairments, it did reduce the rate of motor impairment at 11 years of age [53].

Aimed at determining the pharmacokinetics, safety, and effectiveness of caffeine citrate as an adjuvant therapy with TH, the caffeine citrate clinical trial (NCT03913221; Table 1) recently published some results. Participants received an initial loading dose of 20 mg/kg within 24-hour of birth, followed by two separate daily doses of either 5 mg/kg or 10 mg/kg administered at 24 hour intervals. Each participant received a total of three doses. Despite the limitation of a small cohort, the results suggest that caffeine citrate is well tolerated in this population, with minimal adverse side effects, none of which were drug-related [57].

## Autologous stem cells

Umbilical cord blood (UCB) and umbilical cord tissue (UCT) contain multiple cell types, including hematopoietic stem and progenitor cells (HSPCs) from the blood component of the cord, and mesenchymal stem/stromal cells (MSCs) located in the cord tissue. The collection of UCB and UCT is non-invasive and safe, posing no risk to the mother or baby. The placenta, umbilical cord, and blood are typically considered biological waste, minimizing ethical concerns related to the collection and use of UCB and UCT [58, 59].

UCB- and UCT-derived cells have unique properties that are useful for cell-based therapies. These include a low risk of immune rejection and tumor formation, a good safety profile, and effectiveness in clinical settings [60]. Stem cells from UCB are promising neuroprotective agents due to their ability to cross the BBB, migrate to the site of injury, promote angiogenesis, secrete growth factors, and promote cell proliferation and neurogenesis [61, 62]. Other cell types in UCB aid in neuroprotection by decreasing apoptosis, oxidative stress, and inflammation [62].

Studies have investigated the use of UCB-derived stem cells in treating neurological disorders such as CP, autism, and spinal cord injury. The first human trial involved the use of autologous UCB-derived mononuclear cells (MNCs) to treat CP caused by hypoxia-induced brain damage. The administration of UCB cells aids in functional neuro-regeneration [63].

There are currently two registered clinical trials (NCT02551003 and NCT02881970 – NEOSTEM) investigating the use of UCB as a potential neuroprotective agent. The first trial (NCT02551003) aims to administer autologous UCB, collected at birth, in three doses over the first three days of life, enrolling only babies with severe HIE. The second trial (NCT02881970 – NEOSTEM) focuses on the MNC fraction of UCB only. Autologous MNCs will be administered to babies via injection, using per kilogram dosing. The main challenges with stem cell therapy include determining the optimal timing of treatment and dosing parameters. Both clinical trials aim to conclude in 2025, and their results will hopefully address these challenges.

## Epoetin alfa/erythropoietin

Erythropoietin (EPO) is a cytokine that is predominantly produced by the kidneys in response to hypoxia and plays a role in erythropoiesis within the bone marrow [64]. Epoetin alfa is a commercial recombinant human form of EPO that was initially used for the treatment of anemia [65].

EPO has been recognized as a potential neuroprotective agent as a result of its anti-apoptotic, anti-oxidative, and anti-inflammatory properties. Once EPO binds to its receptor (EPOR), it triggers the phosphorylation of Janus kinase (JAK)-2, which then activates nuclear factor (NF)-κB, signal transducer activator of transcription 5 (STAT5), and phosphatidylinositol-3 kinase (PI3-K) pathways. NF-kB activation contributes to the anti-oxidant properties of EPO by inducing the transcription of SODs and enhances the anti-apoptotic properties of EPO through p53 upregulation [66, 67]. STAT5 activation contributes to the anti-apoptotic effects of EPO by activating the B-cell lymphoma (*BCL*)*-xL* and *BCL-2* genes. Activation of the PI3-K pathway reduces caspase activity. The anti-inflammatory properties of EPO are linked to the reduction in neuronal cell death following the HI event, rather than direct effects on EPOR-expressing immune cells [68]. Although there is evidence to suggest that EPO has neuroprotective properties [69–71], the recently published results of the High-Dose Erythropoietin for Asphyxia and Encephalopathy (HEAL) trial (NCT02811263) did not demonstrate a neuroprotective effect [72]. The HEAL trial will be discussed in the “Unsuccessful clinical trials” section below.

Currently, the Erythropoietin for Hypoxic Ischaemic Encephalopathy in Newborns (PAEAN) trial (NCT03079167; Table 1) is testing the efficacy of epoetin alfa (1000 U/kg; five doses) in combination with TH. It is a phase 3, double-blind, placebo-controlled trial which aims to treat 150 babies. The results of this trial are still pending.

## Allopurinol

Allopurinol is a drug that is indicated for the treatment of gout and acts by inhibiting the activity of xanthine oxidase, an enzyme involved in the production of uric acid. By inhibiting this enzyme, allopurinol reduces the levels of uric acid in the blood [73].

During a HI event, there is an increase in hypoxanthine due to ATP degradation. Hypoxanthine combined with oxygen during reperfusion leads to the formation of the superoxide (O_2_^−^) anion. The reaction is mediated by the xanthine oxidase enzyme, which is inhibited by allopurinol [74, 75].

The Allopurinol in addition to TH treatment for hypoxic-ischemic Brain Injury on Neurocognitive Outcome (ALBINO) trial (NCT03162653); Table 1 is currently investigating the use of allopurinol as a neuroprotective agent for babies with NESHIE, due to its ability to reduce ROS, specifically the superoxide (O_2_^−^) ion. The trial will test the efficacy and safety of allopurinol in combination with TH. A total of 846 babies are expected to be enrolled in the clinical trial, of which half will receive two doses of allopurinol intravenously [76]. The results of the trial are expected in 2025.

## Unsuccessful clinical trials

The overall success rate of clinical drug trials is estimated to be between 10% and 20% [77]. The success rate of phase 1, 2, 3, and the regulatory review are estimated to be 63%, 31%, 58%, and 85%, respectively [78]. It is therefore essential for pharmaceutical companies to choose a drug that is most likely to succeed. There are two primary reasons for drug failures: lack of efficacy, accounting for 40%–50% of failures, and unmanageable toxicity, accounting for 30% of failures [79].

Examples of neuroprotective agents that have failed in clinical trials due to lack of efficacy include EPO, magnesium sulfate, and xenon [72]. The HEAL trial was a phase 3, multi-center, double-blind, randomized, placebo-controlled trial. A total of 257 babies received five doses of epoetin alfa (1000 U/kg) in combination with TH. Despite promising results from phase 1 [80] and 2 [81] trials, the phase 3 trial concluded that EPO does not reduce the risk of death or neurological disability at 22–26 months of age. In addition, the administration of EPO was associated with a higher incidence of adverse events [72]. The authors postulated that TH and EPO may provide equal benefits, which could explain why EPO was unable to confer additional neuroprotective benefits. The dosage and timing of EPO administration also need to be re-evaluated. Even though EPO failed to show efficacy in the HEAL trial, it is still being investigated in the PAEAN trial as an adjuvant therapy for babies with HIE (NCT03079167), with study results pending.

Both trials (HEAL and PAEAN) were designed as randomized, placebo-controlled trials to investigate the neuroprotective effects of EPO; the differences between the two trials are summarised in Table 2.

**Table 2:** Comparison of HEAL and PAEAN clinical trials

Clinical trial	Target population	Population size	Primary outcomes	Secondary outcomes	Reference
HEAL	Term and near term babies (≥ 36 wk gestational age) + TH	500	Death or moderate/severe disability at two years of age	Presence of CP, severity of motor impairment, Bayley scores, epilepsy, and behavioural abnormalities at the age of two years	73
PAEAN	Term and near term (≥ 35 wk gestational age) babies + TH	313		Measures of cognitive function, motor function, need for respiratory and nutritional support, and visual and hearing impairments	NA

*CP* Cerebral palsy, *HEAL* high-dose erythropoietin for asphyxia and encephalopathy, *PAEAN* erythropoietin for hypoxic ischaemic encephalopathy in newborns, *TH* therapeutic hypothermia, *NA* not available

Meta-analyses of smaller randomized clinical trials present confounding views of EPO as a neuroprotective agent. Some studies support EPO as a neuroprotective agent when used as a monotherapy [69–71], while others suggest that EPO is not neuroprotective even in combination with TH [52, 82].

Another neuroprotective agent that has failed to show efficacy in clinical trials is magnesium sulfate (MgSO_4_), a drug commonly used in obstetric medicine [83]. MgSO_4_ functions as an NMDA receptor antagonist, reducing excitotoxic damage, neuronal apoptosis, and necrosis [20, 75, 84]. The safety and tolerability of MgSO_4_ in near-term and term neonates with HIE have been shown, but significant neuroprotective benefits have not been observed [85, 86]. In the study conducted by Gathwala and colleagues, there was a decrease in EEG (43.75% vs. 31.25%) and CT (62.5% vs. 37.5%) abnormalities in the MgSO_4_ + TH group, although these differences were not significant [86]. Given its accessibility, good safety profile, and cost-effectiveness, MgSO_4_ has the potential to be a good neuroprotective agent. However, pre-clinical data and clinical trial results are conflicting [83]. Meta-analyses have suggested some potential short-term in-hospital benefits for the use of MgSO_4_, including a reduction in seizures and improved neurological status at discharge [87]. However, the evidence for these benefits is only moderate and further studies that can demonstrate long-term neuroprotective benefits are required [83]. Xenon, a noble gas used as a volatile anesthetic agent, lacks sufficient efficacy data to support its use as a neuroprotective agent [88]. Xenon readily crosses the BBB and blocks NMDA and AMPA receptors, attenuating excitotoxicity damage in the neonatal brain [89]. Pre-clinical investigations in animal models showed promising results, but a proof-of-concept clinical trial that enrolled 92 babies (TOBY-Xe; NCT 00934700) failed to show improved treatment outcomes in the TH plus Xenon arm [89, 90].

## Discussion

When combining the complexity and high prevalence of HIE in LMICs and the varying effects of TH within these settings with socio-geographic and economic challenges, it becomes clear that more effective and readily available treatments are necessary. Several clinical trials are currently investigating new therapeutic interventions. However, the use of neuroprotective agents in these trials is limited by their administration as adjuvant therapies to TH. Investigations into their neuroprotective effects occur predominantly in high-income countries (HICs), except for the trial in China, which is a middle-income country. There is a need for more large randomized clinical trials within the LMIC setting.

The complex and progressive nature of HIE may respond best to combination therapies with TH; however, stand-alone treatment without TH is likely to be more accessible and feasible in many LMIC settings.

The ideal therapy for HIE in LMICs would be a cost-effective product that is easily accessible, stored and administered, demonstrates efficacy as a stand-alone treatment, and has a wide therapeutic window. If any of the neuroprotective agents currently being investigated show significant efficacy with TH, additional studies will be needed to determine their efficacy as stand-alone treatments without TH in LMIC settings. In addition, the development of such stand-alone treatments would also benefit HIE neonates who might not be able to access or receive TH within 6 h of birth as per current criteria, in both LMIC and HIC settings.

## Conclusions

Disparities in the prevalence of HIE between HICs and LMICs together with variable responses to TH in LMICs underlie the critical importance of studying and contrasting both the pathogenesis and responses to therapy in these two settings. There may be underlying population differences that affect treatment outcomes, which could explain why some research groups see the benefit of TH treatment while the HELIX research group and others did not. These differences are likely to be both constitutional (genetic) and environmental. Understanding these differences could assist in tailoring therapies for different population settings and in the development of novel therapeutic approaches.

## Data Availability

Data sharing not applicable to this article as no datasets were generated or analyzed during the current study. Neonatal encephalopathy due to suspected hypoxic ischemic encephalopathy: pathophysiology, current and emerging treatments
